# Supplementary material for: DMRforPairs: identifying Differentially Methylated Regions between unique samples using array based methylation profiles
Source: BMC Bioinformatics. 2014 May 15;15:141. doi: 10.1186/1471-2105-15-141 (PMC4046028; doi:10.1186/1471-2105-15-141)
Supplement: Additional file 2 — DMRforPairs output for the comparison of A431-MCF7 and NA17018-NA17105. Please start from the HTML files in each folder. Available via the BMC Bioinformatics website. [file 1471-2105-15-141-S2.zip › 1394847754114233_MOESM2_ESM/A431_MCF7/figures/15931.pdf]

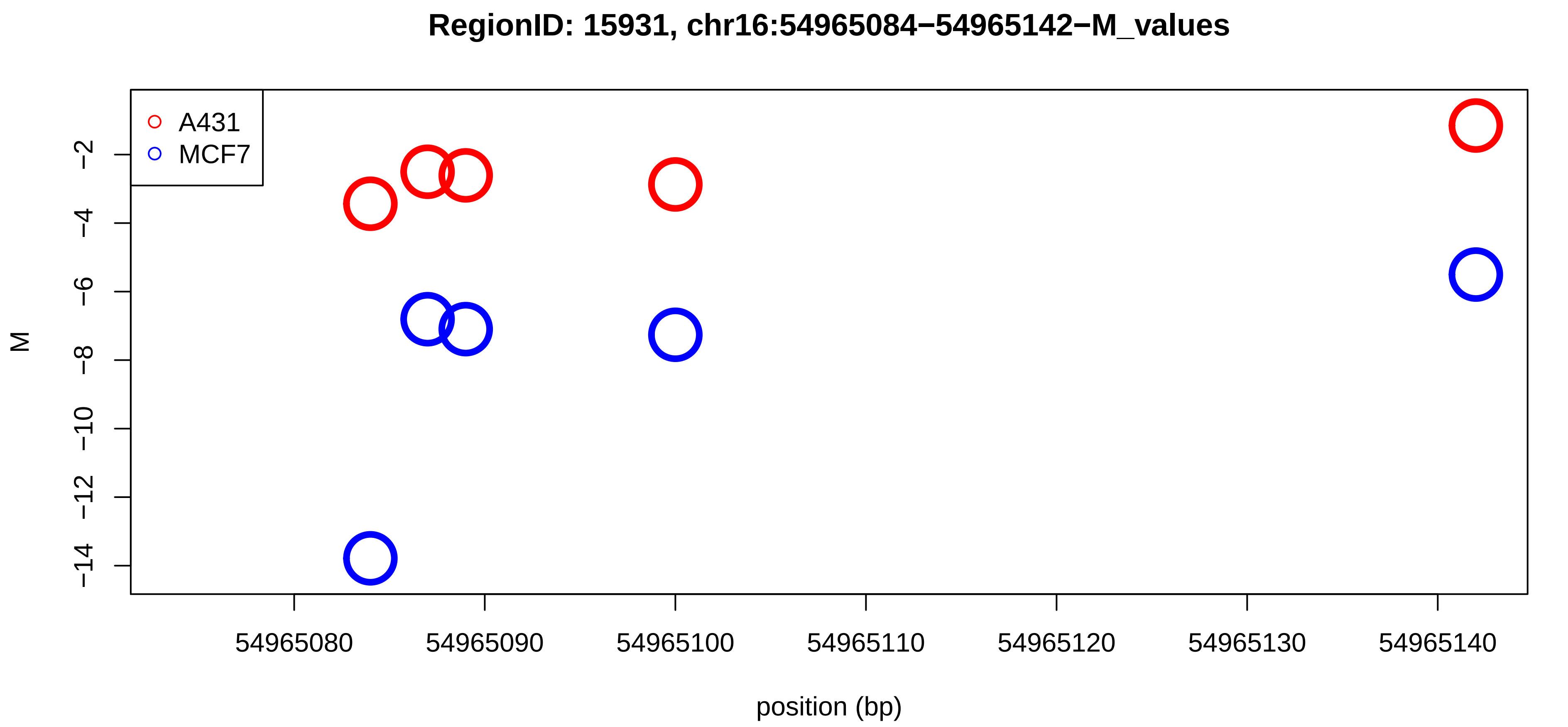
RegionID: 15931, chr16:54965084−54965142−M_values
A431
MCF7
M
−2
−4
−6
−8
−10
−12
−14
54965080
54965090
54965100
54965110
54965120
54965130
54965140
position (bp)

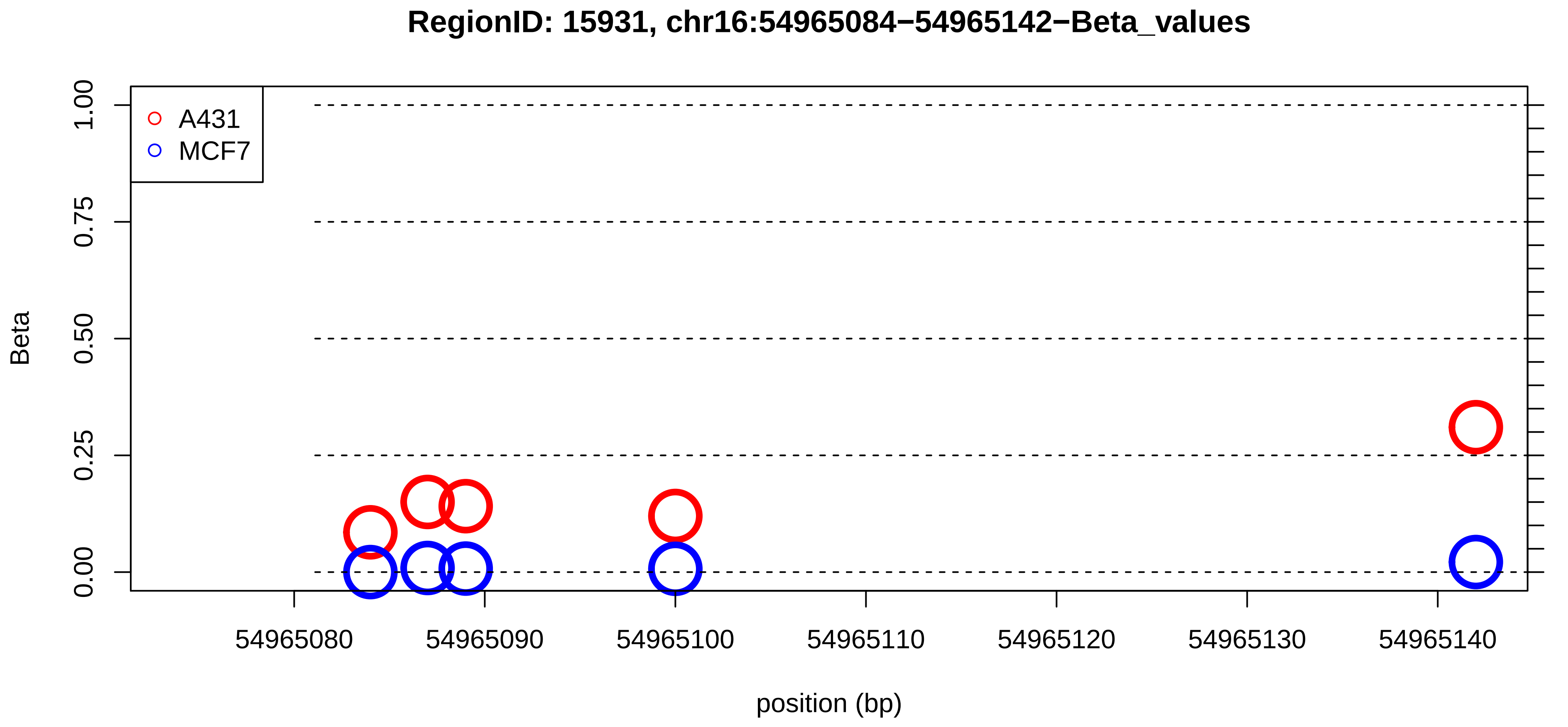
RegionID: 15931, chr16:54965084−54965142−Beta_values
A431
MCF7
Beta
1.00
0.75
0.50
0.25
0.00
54965080
54965090
54965100
54965110
54965120
54965130
54965140
position (bp)

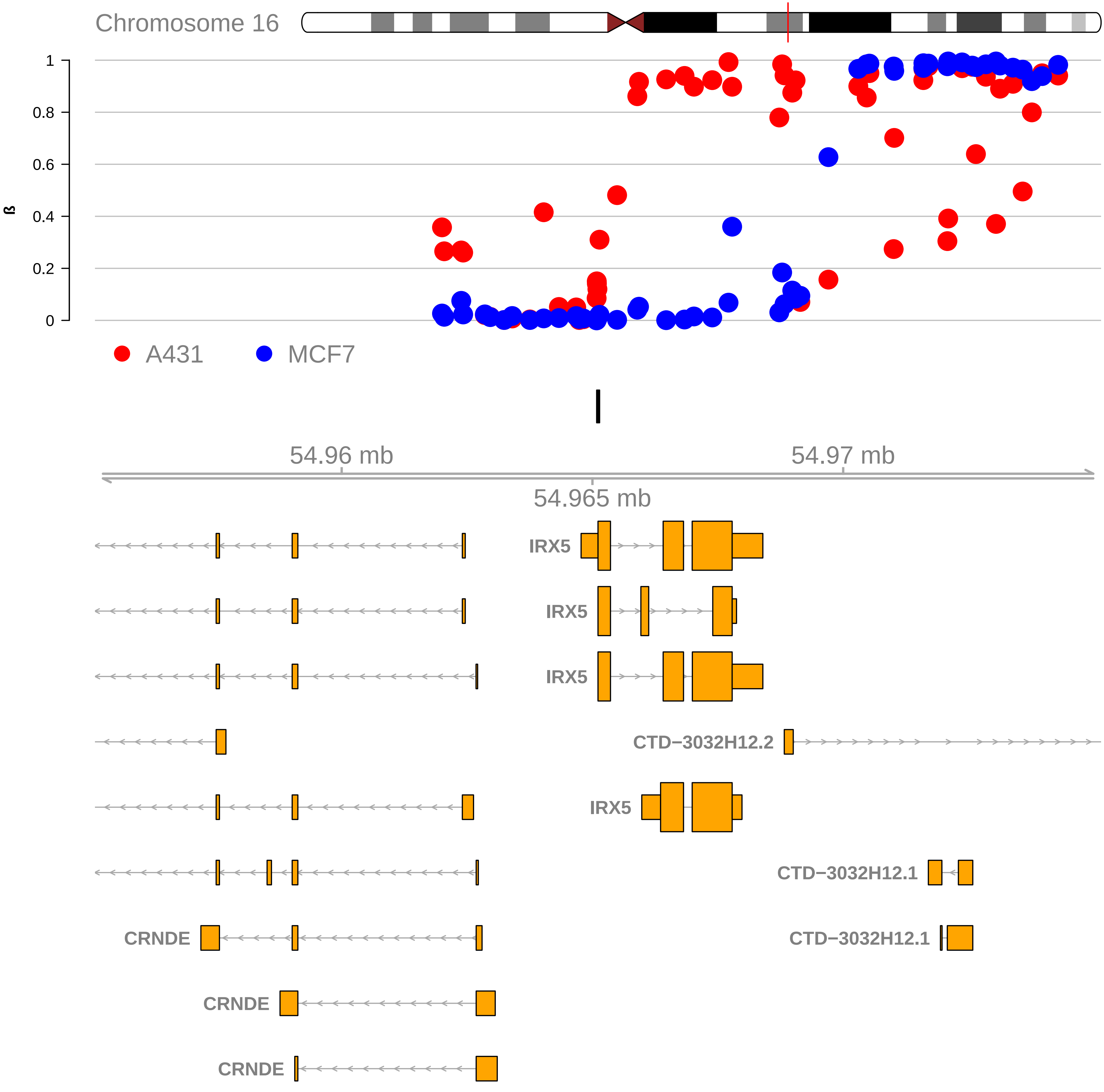

Chromosome 16
1
0.8
0.6
0.4
0.2
0
β
A431
MCF7
54.96 mb
54.97 mb
54.965 mb
IRX5
IRX5
IRX5
CTD−3032H12.2
IRX5
CTD−3032H12.1
CRNDE
CTD−3032H12.1
CRNDE
CRNDE
